# Supplementary material for: Obstructive sleep apnea in obese pregnant women: A prospective study
Source: PLoS One. 2020 Sep 8;15(9):e0238733. doi: 10.1371/journal.pone.0238733 (PMC7478531; doi:10.1371/journal.pone.0238733)
Supplement: S1 Data — (ZIP) [file pone.0238733.s001.zip › 1-DescGlobal.rtf]

DIABETE	Frequency	Percent	Cumulative
Frequency	Cumulative
Percent	
0	69	80.23	69	80.23	
1	17	19.77	86	100.00	


DT1	Frequency	Percent	Cumulative
Frequency	Cumulative
Percent	
0	85	98.84	85	98.84	
1	1	1.16	86	100.00	


DT2	Frequency	Percent	Cumulative
Frequency	Cumulative
Percent	
0	69	80.23	69	80.23	
1	17	19.77	86	100.00	


HTA	Frequency	Percent	Cumulative
Frequency	Cumulative
Percent	
0	68	79.07	68	79.07	
1	18	20.93	86	100.00	


MTEV	Frequency	Percent	Cumulative
Frequency	Cumulative
Percent	
0	72	83.72	72	83.72	
1	14	16.28	86	100.00	


SASFAM	Frequency	Percent	Cumulative
Frequency	Cumulative
Percent	
0	65	75.58	65	75.58	
1	21	24.42	86	100.00	


POLYSOMNO	Frequency	Percent	Cumulative
Frequency	Cumulative
Percent	
0	19	22.09	19	22.09	
1	67	77.91	86	100.00	


SAS	Frequency	Percent	Cumulative
Frequency	Cumulative
Percent	
0	38	56.72	38	56.72	
1	29	43.28	67	100.00	
Frequency Missing = 19	


SASMODERE	Frequency	Percent	Cumulative
Frequency	Cumulative
Percent	
0	42	62.69	42	62.69	
1	25	37.31	67	100.00	
Frequency Missing = 19	


SASSEVERE	Frequency	Percent	Cumulative
Frequency	Cumulative
Percent	
0	63	94.03	63	94.03	
1	4	5.97	67	100.00	
Frequency Missing = 19	


PPC	Frequency	Percent	Cumulative
Frequency	Cumulative
Percent	
0	62	92.54	62	92.54	
1	5	7.46	67	100.00	
Frequency Missing = 19	


DIABETEG	Frequency	Percent	Cumulative
Frequency	Cumulative
Percent	
0	57	66.28	57	66.28	
1	29	33.72	86	100.00	


HOSPIT	Frequency	Percent	Cumulative
Frequency	Cumulative
Percent	
0	31	37.35	31	37.35	
1	52	62.65	83	100.00	
Frequency Missing = 3	


CRITERECOMPOSITE	Frequency	Percent	Cumulative
Frequency	Cumulative
Percent	
0	66	78.57	66	78.57	
1	18	21.43	84	100.00	
Frequency Missing = 2	


SPONT	Frequency	Percent	Cumulative
Frequency	Cumulative
Percent	
0	46	56.10	46	56.10	
1	36	43.90	82	100.00	
Frequency Missing = 4	


DECL	Frequency	Percent	Cumulative
Frequency	Cumulative
Percent	
0	46	56.10	46	56.10	
1	36	43.90	82	100.00	
Frequency Missing = 4	


VB	Frequency	Percent	Cumulative
Frequency	Cumulative
Percent	
0	35	42.68	35	42.68	
1	47	57.32	82	100.00	
Frequency Missing = 4	


CESARIENNE	Frequency	Percent	Cumulative
Frequency	Cumulative
Percent	
0	47	56.63	47	56.63	
1	36	43.37	83	100.00	
Frequency Missing = 3	


CESPROG	Frequency	Percent	Cumulative
Frequency	Cumulative
Percent	
0	67	81.71	67	81.71	
1	15	18.29	82	100.00	
Frequency Missing = 4	


CESARURG	Frequency	Percent	Cumulative
Frequency	Cumulative
Percent	
0	75	91.46	75	91.46	
1	7	8.54	82	100.00	
Frequency Missing = 4	


CESARPTRAVAIL	Frequency	Percent	Cumulative
Frequency	Cumulative
Percent	
0	67	81.71	67	81.71	
1	15	18.29	82	100.00	
Frequency Missing = 4	


TRANSFERT	Frequency	Percent	Cumulative
Frequency	Cumulative
Percent	
0	75	91.46	75	91.46	
1	7	8.54	82	100.00	
Frequency Missing = 4	


INTEGRALITEETUDE	Frequency	Percent	Cumulative
Frequency	Cumulative
Percent	
0	19	22.62	19	22.62	
1	65	77.38	84	100.00	
Frequency Missing = 2	

Variable	N	Mean	Std Dev	Minimum	Maximum	Median	Lower Quartile	Upper Quartile	
AGE
GESTITE
PARITE
POIDSDG
TAILLE
BMI
POIDSFG
PRISEPOIDS
AG
POIDSENF
APGAR1
APGAR5
APGAR10
PH	86
86
86
85
85
85
59
59
81
83
82
82
81
80	30.83
1.87
1.13
119.4
167.2
42.66
124.3
6.14
270.9
3258
9.56
9.82
9.95
7.25	5.17
2.30
1.42
19.17
6.75
6.01
18.31
7.92
16.24
730.7
1.33
0.74
0.27
0.09	19.00
0.00
0.00
88.00
154.0
30.93
89.00
-14.0
219.0
1060
2.00
5.00
8.00
7.03	44.00
15.00
7.00
176.0
185.0
55.36
173.0
22.00
292.0
4400
10.00
10.00
10.00
7.45	31.00
1.00
1.00
116.0
167.0
41.43
125.0
7.00
273.0
3400
10.00
10.00
10.00
7.24	28.00
0.00
0.00
105.0
162.0
37.81
109.0
0.00
268.0
2980
10.00
10.00
10.00
7.20	34.00
3.00
2.00
131.0
173.0
48.28
138.0
12.00
282.0
3780
10.00
10.00
10.00
7.31	
