# Supplementary material for: Obstructive sleep apnea in obese pregnant women: A prospective study
Source: PLoS One. 2020 Sep 8;15(9):e0238733. doi: 10.1371/journal.pone.0238733 (PMC7478531; doi:10.1371/journal.pone.0238733)
Supplement: S1 Data — (ZIP) [file pone.0238733.s001.zip › 2-DescParSAS.rtf]

DIABETE	Frequency	Percent	Cumulative
Frequency	Cumulative
Percent	
0	16	84.21	16	84.21	
1	3	15.79	19	100.00	


DT1	Frequency	Percent	Cumulative
Frequency	Cumulative
Percent	
0	19	100.00	19	100.00	


DT2	Frequency	Percent	Cumulative
Frequency	Cumulative
Percent	
0	16	84.21	16	84.21	
1	3	15.79	19	100.00	


HTA	Frequency	Percent	Cumulative
Frequency	Cumulative
Percent	
0	15	78.95	15	78.95	
1	4	21.05	19	100.00	


MTEV	Frequency	Percent	Cumulative
Frequency	Cumulative
Percent	
0	15	78.95	15	78.95	
1	4	21.05	19	100.00	


SASFAM	Frequency	Percent	Cumulative
Frequency	Cumulative
Percent	
0	14	73.68	14	73.68	
1	5	26.32	19	100.00	


POLYSOMNO	Frequency	Percent	Cumulative
Frequency	Cumulative
Percent	
0	19	100.00	19	100.00	


SAS	Frequency	Percent	Cumulative
Frequency	Cumulative
Percent	
					
Frequency Missing = 19	


SASMODERE	Frequency	Percent	Cumulative
Frequency	Cumulative
Percent	
					
Frequency Missing = 19	


SASSEVERE	Frequency	Percent	Cumulative
Frequency	Cumulative
Percent	
					
Frequency Missing = 19	


PPC	Frequency	Percent	Cumulative
Frequency	Cumulative
Percent	
					
Frequency Missing = 19	


DIABETEG	Frequency	Percent	Cumulative
Frequency	Cumulative
Percent	
0	13	68.42	13	68.42	
1	6	31.58	19	100.00	


HOSPIT	Frequency	Percent	Cumulative
Frequency	Cumulative
Percent	
0	11	64.71	11	64.71	
1	6	35.29	17	100.00	
Frequency Missing = 2	


CRITERECOMPOSITE	Frequency	Percent	Cumulative
Frequency	Cumulative
Percent	
0	16	88.89	16	88.89	
1	2	11.11	18	100.00	
Frequency Missing = 1	


SPONT	Frequency	Percent	Cumulative
Frequency	Cumulative
Percent	
0	11	64.71	11	64.71	
1	6	35.29	17	100.00	
Frequency Missing = 2	


DECL	Frequency	Percent	Cumulative
Frequency	Cumulative
Percent	
0	10	58.82	10	58.82	
1	7	41.18	17	100.00	
Frequency Missing = 2	


VB	Frequency	Percent	Cumulative
Frequency	Cumulative
Percent	
0	7	41.18	7	41.18	
1	10	58.82	17	100.00	
Frequency Missing = 2	


CESARIENNE	Frequency	Percent	Cumulative
Frequency	Cumulative
Percent	
0	10	58.82	10	58.82	
1	7	41.18	17	100.00	
Frequency Missing = 2	


CESPROG	Frequency	Percent	Cumulative
Frequency	Cumulative
Percent	
0	12	70.59	12	70.59	
1	5	29.41	17	100.00	
Frequency Missing = 2	


CESARURG	Frequency	Percent	Cumulative
Frequency	Cumulative
Percent	
0	16	94.12	16	94.12	
1	1	5.88	17	100.00	
Frequency Missing = 2	


CESARPTRAVAIL	Frequency	Percent	Cumulative
Frequency	Cumulative
Percent	
0	16	94.12	16	94.12	
1	1	5.88	17	100.00	
Frequency Missing = 2	


TRANSFERT	Frequency	Percent	Cumulative
Frequency	Cumulative
Percent	
0	17	100.00	17	100.00	
Frequency Missing = 2	


INTEGRALITEETUDE	Frequency	Percent	Cumulative
Frequency	Cumulative
Percent	
0	19	100.00	19	100.00	

DIABETE	Frequency	Percent	Cumulative
Frequency	Cumulative
Percent	
0	32	84.21	32	84.21	
1	6	15.79	38	100.00	


DT1	Frequency	Percent	Cumulative
Frequency	Cumulative
Percent	
0	37	97.37	37	97.37	
1	1	2.63	38	100.00	


DT2	Frequency	Percent	Cumulative
Frequency	Cumulative
Percent	
0	32	84.21	32	84.21	
1	6	15.79	38	100.00	


HTA	Frequency	Percent	Cumulative
Frequency	Cumulative
Percent	
0	35	92.11	35	92.11	
1	3	7.89	38	100.00	


MTEV	Frequency	Percent	Cumulative
Frequency	Cumulative
Percent	
0	33	86.84	33	86.84	
1	5	13.16	38	100.00	


SASFAM	Frequency	Percent	Cumulative
Frequency	Cumulative
Percent	
0	29	76.32	29	76.32	
1	9	23.68	38	100.00	


POLYSOMNO	Frequency	Percent	Cumulative
Frequency	Cumulative
Percent	
1	38	100.00	38	100.00	


SAS	Frequency	Percent	Cumulative
Frequency	Cumulative
Percent	
0	38	100.00	38	100.00	


SASMODERE	Frequency	Percent	Cumulative
Frequency	Cumulative
Percent	
0	38	100.00	38	100.00	


SASSEVERE	Frequency	Percent	Cumulative
Frequency	Cumulative
Percent	
0	38	100.00	38	100.00	


PPC	Frequency	Percent	Cumulative
Frequency	Cumulative
Percent	
0	38	100.00	38	100.00	


DIABETEG	Frequency	Percent	Cumulative
Frequency	Cumulative
Percent	
0	29	76.32	29	76.32	
1	9	23.68	38	100.00	


HOSPIT	Frequency	Percent	Cumulative
Frequency	Cumulative
Percent	
0	12	32.43	12	32.43	
1	25	67.57	37	100.00	
Frequency Missing = 1	


CRITERECOMPOSITE	Frequency	Percent	Cumulative
Frequency	Cumulative
Percent	
0	31	83.78	31	83.78	
1	6	16.22	37	100.00	
Frequency Missing = 1	


SPONT	Frequency	Percent	Cumulative
Frequency	Cumulative
Percent	
0	15	40.54	15	40.54	
1	22	59.46	37	100.00	
Frequency Missing = 1	


DECL	Frequency	Percent	Cumulative
Frequency	Cumulative
Percent	
0	24	64.86	24	64.86	
1	13	35.14	37	100.00	
Frequency Missing = 1	


VB	Frequency	Percent	Cumulative
Frequency	Cumulative
Percent	
0	13	35.14	13	35.14	
1	24	64.86	37	100.00	
Frequency Missing = 1	


CESARIENNE	Frequency	Percent	Cumulative
Frequency	Cumulative
Percent	
0	24	64.86	24	64.86	
1	13	35.14	37	100.00	
Frequency Missing = 1	


CESPROG	Frequency	Percent	Cumulative
Frequency	Cumulative
Percent	
0	33	91.67	33	91.67	
1	3	8.33	36	100.00	
Frequency Missing = 2	


CESARURG	Frequency	Percent	Cumulative
Frequency	Cumulative
Percent	
0	34	94.44	34	94.44	
1	2	5.56	36	100.00	
Frequency Missing = 2	


CESARPTRAVAIL	Frequency	Percent	Cumulative
Frequency	Cumulative
Percent	
0	27	75.00	27	75.00	
1	9	25.00	36	100.00	
Frequency Missing = 2	


TRANSFERT	Frequency	Percent	Cumulative
Frequency	Cumulative
Percent	
0	33	91.67	33	91.67	
1	3	8.33	36	100.00	
Frequency Missing = 2	


INTEGRALITEETUDE	Frequency	Percent	Cumulative
Frequency	Cumulative
Percent	
1	36	100.00	36	100.00	
Frequency Missing = 2	

DIABETE	Frequency	Percent	Cumulative
Frequency	Cumulative
Percent	
0	21	72.41	21	72.41	
1	8	27.59	29	100.00	


DT1	Frequency	Percent	Cumulative
Frequency	Cumulative
Percent	
0	29	100.00	29	100.00	


DT2	Frequency	Percent	Cumulative
Frequency	Cumulative
Percent	
0	21	72.41	21	72.41	
1	8	27.59	29	100.00	


HTA	Frequency	Percent	Cumulative
Frequency	Cumulative
Percent	
0	18	62.07	18	62.07	
1	11	37.93	29	100.00	


MTEV	Frequency	Percent	Cumulative
Frequency	Cumulative
Percent	
0	24	82.76	24	82.76	
1	5	17.24	29	100.00	


SASFAM	Frequency	Percent	Cumulative
Frequency	Cumulative
Percent	
0	22	75.86	22	75.86	
1	7	24.14	29	100.00	


POLYSOMNO	Frequency	Percent	Cumulative
Frequency	Cumulative
Percent	
1	29	100.00	29	100.00	


SAS	Frequency	Percent	Cumulative
Frequency	Cumulative
Percent	
1	29	100.00	29	100.00	


SASMODERE	Frequency	Percent	Cumulative
Frequency	Cumulative
Percent	
0	4	13.79	4	13.79	
1	25	86.21	29	100.00	


SASSEVERE	Frequency	Percent	Cumulative
Frequency	Cumulative
Percent	
0	25	86.21	25	86.21	
1	4	13.79	29	100.00	


PPC	Frequency	Percent	Cumulative
Frequency	Cumulative
Percent	
0	24	82.76	24	82.76	
1	5	17.24	29	100.00	


DIABETEG	Frequency	Percent	Cumulative
Frequency	Cumulative
Percent	
0	15	51.72	15	51.72	
1	14	48.28	29	100.00	


HOSPIT	Frequency	Percent	Cumulative
Frequency	Cumulative
Percent	
0	8	27.59	8	27.59	
1	21	72.41	29	100.00	


CRITERECOMPOSITE	Frequency	Percent	Cumulative
Frequency	Cumulative
Percent	
0	19	65.52	19	65.52	
1	10	34.48	29	100.00	


SPONT	Frequency	Percent	Cumulative
Frequency	Cumulative
Percent	
0	20	71.43	20	71.43	
1	8	28.57	28	100.00	
Frequency Missing = 1	


DECL	Frequency	Percent	Cumulative
Frequency	Cumulative
Percent	
0	12	42.86	12	42.86	
1	16	57.14	28	100.00	
Frequency Missing = 1	


VB	Frequency	Percent	Cumulative
Frequency	Cumulative
Percent	
0	15	53.57	15	53.57	
1	13	46.43	28	100.00	
Frequency Missing = 1	


CESARIENNE	Frequency	Percent	Cumulative
Frequency	Cumulative
Percent	
0	13	44.83	13	44.83	
1	16	55.17	29	100.00	


CESPROG	Frequency	Percent	Cumulative
Frequency	Cumulative
Percent	
0	22	75.86	22	75.86	
1	7	24.14	29	100.00	


CESARURG	Frequency	Percent	Cumulative
Frequency	Cumulative
Percent	
0	25	86.21	25	86.21	
1	4	13.79	29	100.00	


CESARPTRAVAIL	Frequency	Percent	Cumulative
Frequency	Cumulative
Percent	
0	24	82.76	24	82.76	
1	5	17.24	29	100.00	


TRANSFERT	Frequency	Percent	Cumulative
Frequency	Cumulative
Percent	
0	25	86.21	25	86.21	
1	4	13.79	29	100.00	


INTEGRALITEETUDE	Frequency	Percent	Cumulative
Frequency	Cumulative
Percent	
1	29	100.00	29	100.00	

SAS	N Obs	Variable	N	Mean	Std Dev	Minimum	Maximum	Median	Lower Quartile	Upper Quartile	
0	38	AGE
GESTITE
PARITE
POIDSDG
TAILLE
BMI
POIDSFG
PRISEPOIDS
AG
POIDSENF
APGAR1
APGAR5
APGAR10
PH	38
38
38
37
37
37
32
32
37
37
36
36
35
35	29.50
1.45
1.05
115.4
167.2
41.18
120.0
5.38
272.8
3292
9.14
9.58
9.89
7.24	4.75
1.90
1.52
19.67
6.98
5.96
18.38
7.96
14.43
644.7
1.88
1.08
0.40
0.09	22.00
0.00
0.00
90.00
154.0
30.93
89.00
-14.0
231.0
1350
2.00
5.00
8.00
7.04	39.00
8.00
7.00
166.0
185.0
54.92
173.0
22.00
292.0
4400
10.00
10.00
10.00
7.44	29.50
1.00
0.00
110.0
167.0
38.57
117.5
5.00
273.0
3320
10.00
10.00
10.00
7.24	26.00
0.00
0.00
100.0
163.0
37.11
106.0
0.00
268.0
2980
10.00
10.00
10.00
7.19	32.00
3.00
2.00
125.0
172.0
47.75
132.0
10.50
283.0
3690
10.00
10.00
10.00
7.30	
1	29	AGE
GESTITE
PARITE
POIDSDG
TAILLE
BMI
POIDSFG
PRISEPOIDS
AG
POIDSENF
APGAR1
APGAR5
APGAR10
PH	29
29
29
29
29
29
25
25
29
29
29
29
29
29	31.90
2.45
1.24
121.8
166.8
43.84
129.7
7.56
266.9
3165
9.83
10.00
10.00
7.24	4.74
3.10
1.50
17.85
6.75
6.24
17.30
7.91
20.23
937.5
0.54
0.00
0.00
0.10	20.00
0.00
0.00
88.00
155.0
34.38
92.00
-7.00
219.0
1060
8.00
10.00
10.00
7.03	41.00
15.00
5.00
160.0
178.0
55.36
160.0
22.00
290.0
4320
10.00
10.00
10.00
7.45	32.00
2.00
1.00
119.0
167.0
43.13
129.0
10.00
273.0
3240
10.00
10.00
10.00
7.23	30.00
0.00
0.00
114.0
162.0
38.99
122.0
2.00
262.0
2900
10.00
10.00
10.00
7.20	35.00
4.00
2.00
136.0
173.0
49.31
142.0
14.00
281.0
3900
10.00
10.00
10.00
7.28	
