# Supplementary material for: Obstructive sleep apnea in obese pregnant women: A prospective study
Source: PLoS One. 2020 Sep 8;15(9):e0238733. doi: 10.1371/journal.pone.0238733 (PMC7478531; doi:10.1371/journal.pone.0238733)
Supplement: S1 Data — (ZIP) [file pone.0238733.s001.zip › 3-CompSAS.rtf]

Table of SAS by CRITERECOMPOSITE	
SAS	CRITERECOMPOSITE	
Frequency
Percent
Row Pct
Col Pct	0	1	Total	
0	31
46.97
83.78
62.00	6
9.09
16.22
37.50	37
56.06

	
1	19
28.79
65.52
38.00	10
15.15
34.48
62.50	29
43.94

	
Total	50
75.76	16
24.24	66
100.00	
Frequency Missing = 20	


Statistics for Table of SAS by CRITERECOMPOSITE	


Statistic	DF	Value	Prob	
Chi-Square	1	2.9537	0.0857	
Likelihood Ratio Chi-Square	1	2.9469	0.0860	
Continuity Adj. Chi-Square	1	2.0428	0.1529	
Mantel-Haenszel Chi-Square	1	2.9089	0.0881	
Phi Coefficient		0.2115		
Contingency Coefficient		0.2070		
Cramer's V		0.2115		


Fisher's Exact Test	
Cell (1,1) Frequency (F)	31	
Left-sided Pr <= F	0.9777	
Right-sided Pr >= F	0.0768	
		
Table Probability (P)	0.0544	
Two-sided Pr <= P	0.1466	

Effective Sample Size = 66
Frequency Missing = 20	

WARNING: 23% of the data are missing.	


Table of SAS by HTA	
SAS	HTA	
Frequency
Percent
Row Pct
Col Pct	0	1	Total	
0	35
52.24
92.11
66.04	3
4.48
7.89
21.43	38
56.72

	
1	18
26.87
62.07
33.96	11
16.42
37.93
78.57	29
43.28

	
Total	53
79.10	14
20.90	67
100.00	
Frequency Missing = 19	


Statistics for Table of SAS by HTA	


Statistic	DF	Value	Prob	
Chi-Square	1	8.9773	0.0027	
Likelihood Ratio Chi-Square	1	9.1977	0.0024	
Continuity Adj. Chi-Square	1	7.2521	0.0071	
Mantel-Haenszel Chi-Square	1	8.8433	0.0029	
Phi Coefficient		0.3660		
Contingency Coefficient		0.3437		
Cramer's V		0.3660		


Fisher's Exact Test	
Cell (1,1) Frequency (F)	35	
Left-sided Pr <= F	0.9996	
Right-sided Pr >= F	0.0034	
		
Table Probability (P)	0.0030	
Two-sided Pr <= P	0.0051	

Effective Sample Size = 67
Frequency Missing = 19	

WARNING: 22% of the data are missing.	


Table of SAS by DIABETE	
SAS	DIABETE	
Frequency
Percent
Row Pct
Col Pct	0	1	Total	
0	32
47.76
84.21
60.38	6
8.96
15.79
42.86	38
56.72

	
1	21
31.34
72.41
39.62	8
11.94
27.59
57.14	29
43.28

	
Total	53
79.10	14
20.90	67
100.00	
Frequency Missing = 19	


Statistics for Table of SAS by DIABETE	


Statistic	DF	Value	Prob	
Chi-Square	1	1.3848	0.2393	
Likelihood Ratio Chi-Square	1	1.3738	0.2412	
Continuity Adj. Chi-Square	1	0.7630	0.3824	
Mantel-Haenszel Chi-Square	1	1.3641	0.2428	
Phi Coefficient		0.1438		
Contingency Coefficient		0.1423		
Cramer's V		0.1438		


Fisher's Exact Test	
Cell (1,1) Frequency (F)	32	
Left-sided Pr <= F	0.9301	
Right-sided Pr >= F	0.1909	
		
Table Probability (P)	0.1211	
Two-sided Pr <= P	0.3635	

Effective Sample Size = 67
Frequency Missing = 19	

WARNING: 22% of the data are missing.	


Table of SAS by SASFAM	
SAS	SASFAM	
Frequency
Percent
Row Pct
Col Pct	0	1	Total	
0	29
43.28
76.32
56.86	9
13.43
23.68
56.25	38
56.72

	
1	22
32.84
75.86
43.14	7
10.45
24.14
43.75	29
43.28

	
Total	51
76.12	16
23.88	67
100.00	
Frequency Missing = 19	


Statistics for Table of SAS by SASFAM	


Statistic	DF	Value	Prob	
Chi-Square	1	0.0019	0.9656	
Likelihood Ratio Chi-Square	1	0.0019	0.9656	
Continuity Adj. Chi-Square	1	0.0000	1.0000	
Mantel-Haenszel Chi-Square	1	0.0018	0.9658	
Phi Coefficient		0.0053		
Contingency Coefficient		0.0053		
Cramer's V		0.0053		


Fisher's Exact Test	
Cell (1,1) Frequency (F)	29	
Left-sided Pr <= F	0.6321	
Right-sided Pr >= F	0.5943	
		
Table Probability (P)	0.2264	
Two-sided Pr <= P	1.0000	

Effective Sample Size = 67
Frequency Missing = 19	

WARNING: 22% of the data are missing.	


Table of SAS by DIABETEG	
SAS	DIABETEG	
Frequency
Percent
Row Pct
Col Pct	0	1	Total	
0	29
43.28
76.32
65.91	9
13.43
23.68
39.13	38
56.72

	
1	15
22.39
51.72
34.09	14
20.90
48.28
60.87	29
43.28

	
Total	44
65.67	23
34.33	67
100.00	
Frequency Missing = 19	


Statistics for Table of SAS by DIABETEG	


Statistic	DF	Value	Prob	
Chi-Square	1	4.4122	0.0357	
Likelihood Ratio Chi-Square	1	4.4160	0.0356	
Continuity Adj. Chi-Square	1	3.3888	0.0656	
Mantel-Haenszel Chi-Square	1	4.3463	0.0371	
Phi Coefficient		0.2566		
Contingency Coefficient		0.2486		
Cramer's V		0.2566		


Fisher's Exact Test	
Cell (1,1) Frequency (F)	29	
Left-sided Pr <= F	0.9909	
Right-sided Pr >= F	0.0329	
		
Table Probability (P)	0.0238	
Two-sided Pr <= P	0.0422	

Effective Sample Size = 67
Frequency Missing = 19	

WARNING: 22% of the data are missing.	

Wilcoxon Scores (Rank Sums) for Variable PH
Classified by Variable SAS	
SAS	N	Sum of
Scores	Expected
Under H0	Std Dev
Under H0	Mean
Score	
0	35	1139.0	1137.50	74.043466	32.542857	
1	29	941.0	942.50	74.043466	32.448276	
Average scores were used for ties.	


Wilcoxon Two-Sample Test	
Statistic	941.0000	
		
Normal Approximation		
Z	-0.0135	
One-Sided Pr <  Z	0.4946	
Two-Sided Pr > |Z|	0.9892	
		
t Approximation		
One-Sided Pr <  Z	0.4946	
Two-Sided Pr > |Z|	0.9893	
Z includes a continuity correction of 0.5.	


Kruskal-Wallis Test	
Chi-Square	0.0004	
DF	1	
Pr > Chi-Square	0.9838	

Wilcoxon Scores (Rank Sums) for Variable POIDSENF
Classified by Variable SAS	
SAS	N	Sum of
Scores	Expected
Under H0	Std Dev
Under H0	Mean
Score	
0	37	1237.0	1239.50	77.394549	33.432432	
1	29	974.0	971.50	77.394549	33.586207	
Average scores were used for ties.	


Wilcoxon Two-Sample Test	
Statistic	974.0000	
		
Normal Approximation		
Z	0.0258	
One-Sided Pr >  Z	0.4897	
Two-Sided Pr > |Z|	0.9794	
		
t Approximation		
One-Sided Pr >  Z	0.4897	
Two-Sided Pr > |Z|	0.9795	
Z includes a continuity correction of 0.5.	


Kruskal-Wallis Test	
Chi-Square	0.0010	
DF	1	
Pr > Chi-Square	0.9742	

Variable	SAS	N	Mean	Std Dev	Std Err	Minimum	Maximum	
AGE	0	38	29.5000	4.7463	0.7699	22.0000	39.0000	
	1	29	31.8966	4.7385	0.8799	20.0000	41.0000	
	Diff (1-2)		-2.3966	4.7429	1.1695			
PRISEPOIDS	0	32	5.3750	7.9626	1.4076	-14.0000	22.0000	
	1	25	7.5600	7.9061	1.5812	-7.0000	22.0000	
	Diff (1-2)		-2.1850	7.9380	2.1189			


Variable	SAS	Method	Mean	95% CL Mean	Std Dev	95% CL Std Dev	
AGE	0		29.5000	27.9399	31.0601	4.7463	3.8695	6.1405	
	1		31.8966	30.0941	33.6990	4.7385	3.7604	6.4086	
	Diff (1-2)	Pooled	-2.3966	-4.7322	-0.0609	4.7429	4.0493	5.7256	
	Diff (1-2)	Satterthwaite	-2.3966	-4.7350	-0.0581				
PRISEPOIDS	0		5.3750	2.5042	8.2458	7.9626	6.3837	10.5861	
	1		7.5600	4.2965	10.8235	7.9061	6.1733	10.9986	
	Diff (1-2)	Pooled	-2.1850	-6.4313	2.0613	7.9380	6.6923	9.7578	
	Diff (1-2)	Satterthwaite	-2.1850	-6.4333	2.0633				


Variable	Method	Variances	DF	t Value	Pr > |t|	
AGE	Pooled	Equal	65	-2.05	0.0445	
	Satterthwaite	Unequal	60.467	-2.05	0.0447	
PRISEPOIDS	Pooled	Equal	55	-1.03	0.3070	
	Satterthwaite	Unequal	51.884	-1.03	0.3068	


Equality of Variances	
Variable	Method	Num DF	Den DF	F Value	Pr > F	
AGE	Folded F	37	28	1.00	1.0000	
PRISEPOIDS	Folded F	31	24	1.01	0.9838	

Wilcoxon Scores (Rank Sums) for Variable PARITE
Classified by Variable SAS	
SAS	N	Sum of
Scores	Expected
Under H0	Std Dev
Under H0	Mean
Score	
0	38	1244.0	1292.0	73.675878	32.736842	
1	29	1034.0	986.0	73.675878	35.655172	
Average scores were used for ties.	


Wilcoxon Two-Sample Test	
Statistic	1034.0000	
		
Normal Approximation		
Z	0.6447	
One-Sided Pr >  Z	0.2596	
Two-Sided Pr > |Z|	0.5191	
		
t Approximation		
One-Sided Pr >  Z	0.2607	
Two-Sided Pr > |Z|	0.5213	
Z includes a continuity correction of 0.5.	


Kruskal-Wallis Test	
Chi-Square	0.4245	
DF	1	
Pr > Chi-Square	0.5147	

Wilcoxon Scores (Rank Sums) for Variable BMI
Classified by Variable SAS	
SAS	N	Sum of
Scores	Expected
Under H0	Std Dev
Under H0	Mean
Score	
0	37	1084.0	1239.50	77.398588	29.297297	
1	29	1127.0	971.50	77.398588	38.862069	
Average scores were used for ties.	


Wilcoxon Two-Sample Test	
Statistic	1127.0000	
		
Normal Approximation		
Z	2.0026	
One-Sided Pr >  Z	0.0226	
Two-Sided Pr > |Z|	0.0452	
		
t Approximation		
One-Sided Pr >  Z	0.0247	
Two-Sided Pr > |Z|	0.0494	
Z includes a continuity correction of 0.5.	


Kruskal-Wallis Test	
Chi-Square	4.0364	
DF	1	
Pr > Chi-Square	0.0445	
