# Supplementary material for: Obstructive sleep apnea in obese pregnant women: A prospective study
Source: PLoS One. 2020 Sep 8;15(9):e0238733. doi: 10.1371/journal.pone.0238733 (PMC7478531; doi:10.1371/journal.pone.0238733)
Supplement: S1 Data — (ZIP) [file pone.0238733.s001.zip › 4-CompPolysomno.rtf]

Table of POLYSOMNO by CRITERECOMPOSITE	
POLYSOMNO	CRITERECOMPOSITE	
Frequency
Percent
Row Pct
Col Pct	0	1	Total	
0	16
19.05
88.89
24.24	2
2.38
11.11
11.11	18
21.43

	
1	50
59.52
75.76
75.76	16
19.05
24.24
88.89	66
78.57

	
Total	66
78.57	18
21.43	84
100.00	
Frequency Missing = 2	


Statistics for Table of POLYSOMNO by CRITERECOMPOSITE	


Statistic	DF	Value	Prob	
Chi-Square	1	1.4484	0.2288	
Likelihood Ratio Chi-Square	1	1.6222	0.2028	
Continuity Adj. Chi-Square	1	0.7735	0.3791	
Mantel-Haenszel Chi-Square	1	1.4312	0.2316	
Phi Coefficient		0.1313		
Contingency Coefficient		0.1302		
Cramer's V		0.1313		
WARNING: 25% of the cells have expected counts less
than 5. Chi-Square may not be a valid test.	


Fisher's Exact Test	
Cell (1,1) Frequency (F)	16	
Left-sided Pr <= F	0.9452	
Right-sided Pr >= F	0.1925	
		
Table Probability (P)	0.1376	
Two-sided Pr <= P	0.3364	

Effective Sample Size = 84
Frequency Missing = 2	


Table of POLYSOMNO by HTA	
POLYSOMNO	HTA	
Frequency
Percent
Row Pct
Col Pct	0	1	Total	
0	15
17.44
78.95
22.06	4
4.65
21.05
22.22	19
22.09

	
1	53
61.63
79.10
77.94	14
16.28
20.90
77.78	67
77.91

	
Total	68
79.07	18
20.93	86
100.00	


Statistics for Table of POLYSOMNO by HTA	


Statistic	DF	Value	Prob	
Chi-Square	1	0.0002	0.9881	
Likelihood Ratio Chi-Square	1	0.0002	0.9882	
Continuity Adj. Chi-Square	1	0.0000	1.0000	
Mantel-Haenszel Chi-Square	1	0.0002	0.9882	
Phi Coefficient		-0.0016		
Contingency Coefficient		0.0016		
Cramer's V		-0.0016		
WARNING: 25% of the cells have expected counts less
than 5. Chi-Square may not be a valid test.	


Fisher's Exact Test	
Cell (1,1) Frequency (F)	15	
Left-sided Pr <= F	0.6063	
Right-sided Pr >= F	0.6423	
		
Table Probability (P)	0.2486	
Two-sided Pr <= P	1.0000	

Sample Size = 86	


Table of POLYSOMNO by DIABETE	
POLYSOMNO	DIABETE	
Frequency
Percent
Row Pct
Col Pct	0	1	Total	
0	16
18.60
84.21
23.19	3
3.49
15.79
17.65	19
22.09

	
1	53
61.63
79.10
76.81	14
16.28
20.90
82.35	67
77.91

	
Total	69
80.23	17
19.77	86
100.00	


Statistics for Table of POLYSOMNO by DIABETE	


Statistic	DF	Value	Prob	
Chi-Square	1	0.2433	0.6218	
Likelihood Ratio Chi-Square	1	0.2534	0.6147	
Continuity Adj. Chi-Square	1	0.0279	0.8674	
Mantel-Haenszel Chi-Square	1	0.2405	0.6238	
Phi Coefficient		0.0532		
Contingency Coefficient		0.0531		
Cramer's V		0.0532		
WARNING: 25% of the cells have expected counts less
than 5. Chi-Square may not be a valid test.	


Fisher's Exact Test	
Cell (1,1) Frequency (F)	16	
Left-sided Pr <= F	0.7893	
Right-sided Pr >= F	0.4489	
		
Table Probability (P)	0.2382	
Two-sided Pr <= P	0.7529	

Sample Size = 86	


Table of POLYSOMNO by SASFAM	
POLYSOMNO	SASFAM	
Frequency
Percent
Row Pct
Col Pct	0	1	Total	
0	14
16.28
73.68
21.54	5
5.81
26.32
23.81	19
22.09

	
1	51
59.30
76.12
78.46	16
18.60
23.88
76.19	67
77.91

	
Total	65
75.58	21
24.42	86
100.00	


Statistics for Table of POLYSOMNO by SASFAM	


Statistic	DF	Value	Prob	
Chi-Square	1	0.0476	0.8274	
Likelihood Ratio Chi-Square	1	0.0470	0.8284	
Continuity Adj. Chi-Square	1	0.0000	1.0000	
Mantel-Haenszel Chi-Square	1	0.0470	0.8284	
Phi Coefficient		-0.0235		
Contingency Coefficient		0.0235		
Cramer's V		-0.0235		
WARNING: 25% of the cells have expected counts less
than 5. Chi-Square may not be a valid test.	


Fisher's Exact Test	
Cell (1,1) Frequency (F)	14	
Left-sided Pr <= F	0.5216	
Right-sided Pr >= F	0.7056	
		
Table Probability (P)	0.2273	
Two-sided Pr <= P	1.0000	

Sample Size = 86	


Table of POLYSOMNO by DIABETEG	
POLYSOMNO	DIABETEG	
Frequency
Percent
Row Pct
Col Pct	0	1	Total	
0	13
15.12
68.42
22.81	6
6.98
31.58
20.69	19
22.09

	
1	44
51.16
65.67
77.19	23
26.74
34.33
79.31	67
77.91

	
Total	57
66.28	29
33.72	86
100.00	


Statistics for Table of POLYSOMNO by DIABETEG	


Statistic	DF	Value	Prob	
Chi-Square	1	0.0501	0.8230	
Likelihood Ratio Chi-Square	1	0.0505	0.8223	
Continuity Adj. Chi-Square	1	0.0000	1.0000	
Mantel-Haenszel Chi-Square	1	0.0495	0.8240	
Phi Coefficient		0.0241		
Contingency Coefficient		0.0241		
Cramer's V		0.0241		


Fisher's Exact Test	
Cell (1,1) Frequency (F)	13	
Left-sided Pr <= F	0.6858	
Right-sided Pr >= F	0.5271	
		
Table Probability (P)	0.2130	
Two-sided Pr <= P	1.0000	

Sample Size = 86	

Variable	POLYSOMNO	N	Mean	Std Dev	Std Err	Minimum	Maximum	
PH	0	16	7.2700	0.0837	0.0209	7.0400	7.4200	
	1	64	7.2409	0.0924	0.0115	7.0300	7.4500	
	Diff (1-2)		0.0291	0.0907	0.0254			


Variable	POLYSOMNO	Method	Mean	95% CL Mean	Std Dev	95% CL Std Dev	
PH	0		7.2700	7.2254	7.3146	0.0837	0.0618	0.1295	
	1		7.2409	7.2179	7.2640	0.0924	0.0787	0.1119	
	Diff (1-2)	Pooled	0.0291	-0.0214	0.0796	0.0907	0.0785	0.1076	
	Diff (1-2)	Satterthwaite	0.0291	-0.0201	0.0783				


Variable	Method	Variances	DF	t Value	Pr > |t|	
PH	Pooled	Equal	78	1.15	0.2554	
	Satterthwaite	Unequal	24.979	1.22	0.2352	


Equality of Variances	
Variable	Method	Num DF	Den DF	F Value	Pr > F	
PH	Folded F	63	15	1.22	0.6974	

Wilcoxon Scores (Rank Sums) for Variable POIDSENF
Classified by Variable POLYSOMNO	
POLYSOMNO	N	Sum of
Scores	Expected
Under H0	Std Dev
Under H0	Mean
Score	
0	17	732.50	714.0	88.615355	43.088235	
1	66	2753.50	2772.0	88.615355	41.719697	
Average scores were used for ties.	


Wilcoxon Two-Sample Test	
Statistic	732.5000	
		
Normal Approximation		
Z	0.2031	
One-Sided Pr >  Z	0.4195	
Two-Sided Pr > |Z|	0.8390	
		
t Approximation		
One-Sided Pr >  Z	0.4198	
Two-Sided Pr > |Z|	0.8395	
Z includes a continuity correction of 0.5.	


Kruskal-Wallis Test	
Chi-Square	0.0436	
DF	1	
Pr > Chi-Square	0.8346	

Variable	POLYSOMNO	N	Mean	Std Dev	Std Err	Minimum	Maximum	
AGE	0	19	31.8421	6.1938	1.4209	19.0000	44.0000	
	1	67	30.5373	4.8565	0.5933	20.0000	41.0000	
	Diff (1-2)		1.3048	5.1723	1.3444			
PRISEPOIDS	0	2	0.5000	6.3640	4.5000	-4.0000	5.0000	
	1	57	6.3333	7.9425	1.0520	-14.0000	22.0000	
	Diff (1-2)		-5.8333	7.9175	5.6959			


Variable	POLYSOMNO	Method	Mean	95% CL Mean	Std Dev	95% CL Std Dev	
AGE	0		31.8421	28.8568	34.8274	6.1938	4.6801	9.1595	
	1		30.5373	29.3527	31.7219	4.8565	4.1508	5.8535	
	Diff (1-2)	Pooled	1.3048	-1.3686	3.9782	5.1723	4.4945	6.0925	
	Diff (1-2)	Satterthwaite	1.3048	-1.8691	4.4786				
PRISEPOIDS	0		0.5000	-56.6779	57.6779	6.3640	2.8393	203.1	
	1		6.3333	4.2259	8.4408	7.9425	6.7055	9.7434	
	Diff (1-2)	Pooled	-5.8333	-17.2392	5.5725	7.9175	6.6935	9.6935	
	Diff (1-2)	Satterthwaite	-5.8333	-52.2458	40.5792				


Variable	Method	Variances	DF	t Value	Pr > |t|	
AGE	Pooled	Equal	84	0.97	0.3345	
	Satterthwaite	Unequal	24.62	0.85	0.4050	
PRISEPOIDS	Pooled	Equal	57	-1.02	0.3101	
	Satterthwaite	Unequal	1.1122	-1.26	0.4101	


Equality of Variances	
Variable	Method	Num DF	Den DF	F Value	Pr > F	
AGE	Folded F	18	66	1.63	0.1575	
PRISEPOIDS	Folded F	56	1	1.56	1.0000	

Wilcoxon Scores (Rank Sums) for Variable PARITE
Classified by Variable POLYSOMNO	
POLYSOMNO	N	Sum of
Scores	Expected
Under H0	Std Dev
Under H0	Mean
Score	
0	19	869.50	826.50	90.335126	45.763158	
1	67	2871.50	2914.50	90.335126	42.858209	
Average scores were used for ties.	


Wilcoxon Two-Sample Test	
Statistic	869.5000	
		
Normal Approximation		
Z	0.4705	
One-Sided Pr >  Z	0.3190	
Two-Sided Pr > |Z|	0.6380	
		
t Approximation		
One-Sided Pr >  Z	0.3196	
Two-Sided Pr > |Z|	0.6392	
Z includes a continuity correction of 0.5.	


Kruskal-Wallis Test	
Chi-Square	0.2266	
DF	1	
Pr > Chi-Square	0.6341	

Wilcoxon Scores (Rank Sums) for Variable BMI
Classified by Variable POLYSOMNO	
POLYSOMNO	N	Sum of
Scores	Expected
Under H0	Std Dev
Under H0	Mean
Score	
0	19	918.50	817.0	94.797936	48.342105	
1	66	2736.50	2838.0	94.797936	41.462121	
Average scores were used for ties.	


Wilcoxon Two-Sample Test	
Statistic	918.5000	
		
Normal Approximation		
Z	1.0654	
One-Sided Pr >  Z	0.1433	
Two-Sided Pr > |Z|	0.2867	
		
t Approximation		
One-Sided Pr >  Z	0.1449	
Two-Sided Pr > |Z|	0.2897	
Z includes a continuity correction of 0.5.	


Kruskal-Wallis Test	
Chi-Square	1.1464	
DF	1	
Pr > Chi-Square	0.2843	

POLYSOMNO	N Obs	Variable	N	Mean	Std Dev	Minimum	Maximum	Median	Lower Quartile	Upper Quartile	
0	19	POIDSENF
PH
AGE
PARITE
BMI
PRISEPOIDS	17
16
19
19
19
2	3340
7.27
31.84
1.11
43.74
0.50	487.6
0.08
6.19
1.10
5.40
6.36	2060
7.04
19.00
0.00
36.33
-4.00	4000
7.42
44.00
4.00
54.32
5.00	3500
7.26
32.00
1.00
42.45
0.50	3040
7.24
27.00
0.00
40.01
-4.00	3635
7.32
37.00
2.00
46.49
5.00	
1	67	POIDSENF
PH
AGE
PARITE
BMI
PRISEPOIDS	66
64
67
67
66
57	3236
7.24
30.54
1.13
42.35
6.33	782.9
0.09
4.86
1.51
6.18
7.94	1060
7.03
20.00
0.00
30.93
-14.0	4400
7.45
41.00
7.00
55.36
22.00	3320
7.24
30.00
1.00
40.40
7.00	2970
7.19
28.00
0.00
37.29
0.00	3820
7.30
34.00
2.00
48.50
12.00	
