# Supplementary material for: Obstructive sleep apnea in obese pregnant women: A prospective study
Source: PLoS One. 2020 Sep 8;15(9):e0238733. doi: 10.1371/journal.pone.0238733 (PMC7478531; doi:10.1371/journal.pone.0238733)
Supplement: S1 Data — (ZIP) [file pone.0238733.s001.zip › 5-DescSASmanq.rtf]

SAS	N Obs	Variable	N	Mean	Std Dev	Minimum	Maximum	Median	Lower Quartile	Upper Quartile	
.	19	AGE
GESTITE
PARITE
POIDSDG
TAILLE
BMI
POIDSFG
PRISEPOIDS
AG
POIDSENF
APGAR1
APGAR5
APGAR10
PH	19
19
19
19
19
19
2
2
15
17
17
17
17
16	31.84
1.84
1.11
123.5
167.8
43.74
125.0
0.50
274.2
3340
10.00
10.00
10.00
7.27	6.19
1.30
1.10
19.62
6.60
5.40
21.21
6.36
9.97
487.6
0.00
0.00
0.00
0.08	19.00
0.00
0.00
94.00
158.0
36.33
110.0
-4.00
254.0
2060
10.00
10.00
10.00
7.04	44.00
4.00
4.00
176.0
180.0
54.32
140.0
5.00
290.0
4000
10.00
10.00
10.00
7.42	32.00
2.00
1.00
123.0
167.0
42.45
125.0
0.50
275.0
3500
10.00
10.00
10.00
7.26	27.00
1.00
0.00
108.0
162.0
40.01
110.0
-4.00
271.0
3040
10.00
10.00
10.00
7.24	37.00
3.00
2.00
140.0
174.0
46.49
140.0
5.00
281.0
3635
10.00
10.00
10.00
7.32	

SAS	Frequency	Percent	Cumulative
Frequency	Cumulative
Percent	
0	38	56.72	38	56.72	
1	29	43.28	67	100.00	
Frequency Missing = 19	


Binomial Proportion	
SAS = 1	
Proportion	0.4328	
ASE	0.0605	
95% Lower Conf Limit	0.3142	
95% Upper Conf Limit	0.5515	
		
Exact Conf Limits		
95% Lower Conf Limit	0.3122	
95% Upper Conf Limit	0.5596	

Test of H0: Proportion = 0.5	
ASE under H0	0.0611	
Z	-1.0995	
One-sided Pr <  Z	0.1358	
Two-sided Pr > |Z|	0.2715	

Effective Sample Size = 67
Frequency Missing = 19	

WARNING: 22% of the data are missing.	

sasnew	Frequency	Percent	Cumulative
Frequency	Cumulative
Percent	
0	57	66.28	57	66.28	
1	29	33.72	86	100.00	


Binomial Proportion	
sasnew = 1	
Proportion	0.3372	
ASE	0.0510	
95% Lower Conf Limit	0.2373	
95% Upper Conf Limit	0.4371	
		
Exact Conf Limits		
95% Lower Conf Limit	0.2388	
95% Upper Conf Limit	0.4472	

Test of H0: Proportion = 0.5	
ASE under H0	0.0539	
Z	-3.0193	
One-sided Pr <  Z	0.0013	
Two-sided Pr > |Z|	0.0025	

Sample Size = 86	
