# Supplementary material for: Obstructive sleep apnea in obese pregnant women: A prospective study
Source: PLoS One. 2020 Sep 8;15(9):e0238733. doi: 10.1371/journal.pone.0238733 (PMC7478531; doi:10.1371/journal.pone.0238733)
Supplement: Attachment — Submitted filename: Response to Reviewers OSA.docx [file pone.0238733.s002.docx]

Reviewers' comments:

Reviewer's Responses to Questions

**Comments to the Author**

**1. Is the manuscript technically sound, and do the data support the conclusions?**

The manuscript must describe a technically sound piece of scientific research with data that supports the conclusions. Experiments must have been conducted rigorously, with appropriate controls, replication, and sample sizes. The conclusions must be drawn appropriately based on the data presented. 

Reviewer #1: Yes

Reviewer #2: Yes

**2. Has the statistical analysis been performed appropriately and rigorously?** 

Reviewer #1: Yes

Reviewer #2: Yes

**3. Have the authors made all data underlying the findings in their manuscript fully available?**

The [PLOS Data policy](http://www.plosone.org/static/policies.action#sharing) requires authors to make all data underlying the findings described in their manuscript fully available without restriction, with rare exception (please refer to the Data Availability Statement in the manuscript PDF file). The data should be provided as part of the manuscript or its supporting information, or deposited to a public repository. For example, in addition to summary statistics, the data points behind means, medians and variance measures should be available. If there are restrictions on publicly sharing data—e.g. participant privacy or use of data from a third party—those must be specified.

Reviewer #1: Yes

Reviewer #2: Yes

**4. Is the manuscript presented in an intelligible fashion and written in standard English?**

PLOS ONE does not copyedit accepted manuscripts, so the language in submitted articles must be clear, correct, and unambiguous. Any typographical or grammatical errors should be corrected at revision, so please note any specific errors here.

Reviewer #1: Yes

Reviewer #2: Yes

**5. Review Comments to the Author**

Please use the space provided to explain your answers to the questions above. You may also include additional comments for the author, including concerns about dual publication, research ethics, or publication ethics. (Please upload your review as an attachment if it exceeds 20,000 characters)

**Reviewer #1:**

The manuscript “Obstructive sleep apnea in obese pregnant women: a prospective study” by Ghesquière et al. included analysis of 67 obese pregnant women, with and without OSA. The authors report the prevalence of OSA in those women, as well as clinical morbidities such as diabetes, hypertension, and effects on the babies. The study is very nicely done, and the results are presented clearly. The authors did a very nice job of addressing other studies with results that differed from their own, as well as pointing out the caveats in their own study. This information is important, and in general, is lacking in the field. The attention to BMI here is an important variable that is usually not well-addressed.

I have just a few minor issues that if addressed, would help to strengthen this manuscript.

1) It would be interesting to and important for others in the field if the authors could indicate which morbidities were present in the same women. For example, was it the same women who had diabetes and chronic hypertension? And was it those women who had vascular complications and had to have C-sections and/or induced labor? This information is important to better understand the consequences of OSA on these outcomes.

Thank you for this suggestion. I added some results (just descriptive) and one sentence in discussion:

In results: “Fourteen (20.9%) had a history of chronic hypertension, 14 (20.9%) of diabetes and 6 (9%) of the two”

“Among these patients who had vascular disease complications, 4 had a history of chronic hypertension (28.6%) and 2 of diabetes (13.3%).”

“Among patients who had cesarean sections, 10/16 (62.5%) in OSA group and 5/13 (38.5%) in non OSA group had a history of chronic hypertension and/or diabetes. Among patients who had induction of labor, 10/16 (62.5%) in OSA group and 6/13 (46.2%) in non OSA group had a history of chronic hypertension and/or diabetes.”

In discussion: “We observed too a trend to more induction and more cesarean section in OSA group, but in these patients, there seemed to have more comorbities”

2) In a similar vein, there appear to be statistical trends in some of the endpoints listed, especially in Table 2, that with additional power, would likely be able to be sussed out. In the Results, one of the "statistical trends" was discussed, but others were not. Often, p values

Thank you for your remark. I changed the sentence: “~~Among the characteristics of delivery,~~ The groups did not differ significantly about characteristics of delivery ~~for rates of vaginal delivery, cesarean delivery, or labor induction (~~*~~P~~*~~>0.05)~~ but there was a trend for more induction (n=16 (57.1%) vs n=13 (35.1%), P=0.08) and more cesarean section (n=16 (55.2%) vs n=13 (35.1%), P=0.10) in OSA group”

3) While the paper is nicely written, there is too much repetition and redundancy throughout. For example, in the Methods, when discussing the gestational diabetes in the Results section on p8, and the 4 women with severe OSA who were offered CPAP treatment.

Many thanks for this suggestion, you’re right. I modified and I removed repetitions :

In the results: ~~The~~ For the second of our secondary objectives, ~~was to study whether various criteria,~~ ~~including age, parity, BMI, history of chronic hypertension, history of diabetes, family history of OSA, weight gain during pregnancy, and gestational diabetes, might be risk factors for developing OSA in this population. A~~ a significant difference was observed between the groups for women's age, prepregnancy history of hypertension, gestational diabetes, and mean BMI (Table 3)

In the discussion: ~~Of the four women with severe OSA, only three agreed to start CPAP treatment and only one continued it to delivery~~.

**Reviewer #2:** In the article under review the authors present a prospective study where they performed inpatient polysomnography sleep testing between 24 and 32 weeks gestation on a cohort of obese pregnant women all with a BMI of >35. They then compared groups with and without OSA and looked at birth outcomes associated with OSA in a cohort of women at a University Hospital in Lille France. The study included a total of 67 women.

Although the authors did perform a power calculation, prior large prospective studies determined sample size with the primary objective of a tool to screen obese pregnant women for OSA. They assumed a 60% detection rate and estimated they needed 182 subjects. I am wondering why the calculation differed so much in this current study with almost half the participants needed? The authors were also underpowered to look at secondary outcomes and should note that. This is an interesting study that will be an important contribution to the literature and is well written.

**Specific comments include:
1) Please review how chronic hypertension was defined as well as the French definitions for preeclampsia :**

Thank you for this remark. I added a reference (N°22) to the recommendations of the French society of arterial hypertension in the references, which defined chronic arterial hypertension and preeclampsia: 22. Consensus d'Experts de la Société Française d'Hypertension Artérielle (SFTA). HTA et Grossesse [Internet]. 2015. Available from: http://www.sfhta.eu/wp-content/uploads/2017/03/ Consensus-dexperts-HTA-et-Grossesse-de-la-SFHTA-D%C3%A9c.-2015.pdf”

**2) Please define what medications were part of the exclusion criteria**

I added types of medications: “or took medication likely to modify OSA (antipsychotics, anxiolytics, hypnotics and sedatives and muscle relaxants).”

**3) The authors should state in the limitations that they were underpowered for the secondary outcomes.**

I added in discussion: “Finally, our observation of trends that did not reach significance for some of the criteria studied (the composite criterion, cesarean deliveries, and labor induction) suggests a lack of power for the secondary outcomes due to an insufficient number of women included.”

**4) FGR as defined as <2500 grams does not account for gestational age. The authors should use either SGA (birthweight <10th percentile for gestational age) or the appropriate in utero estimate**

Thank you for this remark. In our study, FGR was defined as birthweight < 10th percentile for gestational age. I added in the methods: « defined by birthweight <10th percentile for gestational age)”

**5) The authors should consider removing Table 1 and presenting the clinical and demographic factors by study group (OSA versus no OSA) as well as the outcomes by study group**

I removed this table and table 3 is became table 1.

**Table 1: Maternal characteristics for all patients and compared to OSA group and no OSA group to study risk factors for developing an OSA.**

| Maternal characteristics | All patients  (N=67) | OSA group (N=29) | No OSA group  (N=38) | p |
| --- | --- | --- | --- | --- |
| Age (years)* | 30.5 ± 4.9 | 31.9 ± 4.7 | 29.5 ± 4.8 | **0.04** |
| Parity* | 1.1 ± 1.5 | 1.2 ± 1.5 | 1.1 ± 1.5 | 0.52 |
| BMI (kg/m^2^)* | 42.4 ± 6.2 | 43.8 ± 6.2 | 41.2 ± 6 | **0.05** |
| Diabetes (type 1or type 2) ° | 14 (20.9) | 8 (27.6) | 6 (15.8) | 0.24 |
| Chronic hypertension ° | 14 (20.9) | 11 (37.9) | 3 (7.9) | **0.003** |
| Family member with OSA° | 16 (23.9) | 7 (24.1) | 9 (23.7) | 0.97 |
| Gestational diabetes° | 23 (34.3) | 14 (48.3) | 9 (23.7) | **0.04** |
| Weight gain (kg)* | 6.3 ± 7.9 | 7.6 ± 7.9 | 5.4 ± 8 | 0.31 |

*BMI = Body Mass Index; OSA = obstructive sleep apnea.*

*(*) and (°) : Data are on average ± standard deviation* or N (%)°. p < 0.05 is significant.*

**6) The authors should show a flow sheet with their entire population (all women BMI >35) and then exclusion criteria, pregnancy loss, withdrew, delivered elsewhere, did not participate, stillbirths? Periviable deliveries?**

I modified the figure 1. Lots of patients were at more of 24WA at the first consultation, because majority of our patients have just the last consultations (7 months, 8 months and 9 months’s consultations) at the maternity. It was for that it was difficult to have the necessary number of patients for the study.

**Figure 1 : Flow chart :**

**7) Table 3 should be table 1 (maternal characteristics)**

Table 3 became table 1

**Table 1: Maternal characteristics for all patients and compared to OSA group and no OSA group to study risk factors for developing an OSA.**

| Maternal characteristics | All patients  (N=67) | OSA group (N=29) | No OSA group  (N=38) | p |
| --- | --- | --- | --- | --- |
| Age (years)* | 30.5 ± 4.9 | 31.9 ± 4.7 | 29.5 ± 4.8 | **0.04** |
| Parity* | 1.1 ± 1.5 | 1.2 ± 1.5 | 1.1 ± 1.5 | 0.52 |
| BMI (kg/m^2^)* | 42.4 ± 6.2 | 43.8 ± 6.2 | 41.2 ± 6 | **0.05** |
| Diabetes (type 1or type 2) ° | 14 (20.9) | 8 (27.6) | 6 (15.8) | 0.24 |
| Chronic hypertension ° | 14 (20.9) | 11 (37.9) | 3 (7.9) | **0.003** |
| Family member with OSA° | 16 (23.9) | 7 (24.1) | 9 (23.7) | 0.97 |
| Gestational diabetes° | 23 (34.3) | 14 (48.3) | 9 (23.7) | **0.04** |
| Weight gain (kg)* | 6.3 ± 7.9 | 7.6 ± 7.9 | 5.4 ± 8 | 0.31 |

*BMI = Body Mass Index; OSA = obstructive sleep apnea.*

*(*) and (°) : Data are on average ± standard deviation* or N (%)°. p < 0.05 is significant.*

**8) Is there information on race/ethnicity? Smoking? Asthma?**

I don’t have information on race/ethnicity but I have information for smoking and asthma. Just 4 patients smoked and 7 patients had asthma so they did not seem important to us to add this information because of the small number.

**9) Can the authors report gestational weeks of delivery? Preterm birth before 37 weeks? 32 weeks?**

I reported in the table 2 and I added preterm birth before 37weeks and before 32weeks. There was no significant difference between 2 groups.

**Table 2: Characteristics of pregnancy, delivery and new born according to the OSA or no OSA groups.**

|  |  | OSA group (N=29) | No OSA group  (N=38) | p |
| --- | --- | --- | --- | --- |
| Characteristics of pregnancy: | |  |  |  |
|  | Gestational diabetes° | 14 (48.3) | 9 (23.7) | **0.04** |
|  | Weight gain (kg)* | 7.6 ± 7.9 | 5.4 ± 8 | 0.31 |
|  | Hospitalization° | 21 (72.4) | 25 (67.6) | 0.67 |
|  | Vascular complication or disease (composite criterion) ° | 10 (34.5) | 6 (16.2) | 0.09 |
| Characteristics of delivery: | |  |  |  |
|  | Term at delivery (WA)* | 38.1 ± 2.9 | 39 ± 2.1 | 0.36 |
|  | Preterm birth before 37 WA | 6 (20.7) | 5 (13.2) | 0.41 |
|  | Preterm birth before 32 WA | 2 (6.7) | 0 (0) | 0.10 |
|  | Vaginal delivery ° | 13 (46.4) | 24 (64.9) | 0.14 |
|  | Cesarean section° | 16 (55.2) | 13 (35.1) | 0.10 |
|  | Scheduled cesarean section° | 7 (24.1) | 3 (8.3) | 0.10 |
|  | Cesarean section during labor ° | 5 (17.2) | 9 (25) | 0.45 |
|  | Emergency cesarean section ° | 4 (13.8) | 2 (5.6) | 0.39 |
|  | Induced labor° | 16/28 (57.1) | 13 (35.1) | 0.08 |
| Neonatal characteristics: | |  |  |  |
|  | Birth weight (kg)* | 3165 ± 937.5 | 3292 ± 644.7 | 0.98 |
|  | pH status* | 7.24 ± 0.10 | 7.24 ± 0.09 | 0.99 |
|  | NICU transfer° | 4 (13.8) | 3 (8.3) | 0.69 |

*BMI = Body Mass Index; OSA = obstructive sleep apnea; WA = weeks of amenorrhea.*

*(*) and (°) : Data are on average ± standard deviation* or N (%)°. p < 0.05 is significant*
